# Autoimmune Encephalitis Masquerading As Acute Psychosis: A Cause of Delayed Treatment

**DOI:** 10.7759/cureus.105852

**Published:** 2026-03-25

**Authors:** Mansi Sharma, Suhasini Rallabandi, Abhay Sharma, Rahul Kashyap

**Affiliations:** 1 Internal Medicine, University of North Carolina Wayne, Goldsboro, USA; 2 Internal Medicine, Banner Baywood Medical Center, Phoenix, USA; 3 Hospital Medicine, University of North Carolina Wayne, Goldsboro, USA; 4 Medicine, Drexel University College of Medicine, Philadelphia, USA; 5 Research, Global Remote Research Scholars Program, Princeton Junction, USA; 6 Critical Care Medicine, Mayo Clinic, Rochester, USA; 7 Research, WellSpan Health, York, USA

**Keywords:** altered mental status in young, anti nmda receptor encephalitis, autoimmune encephalitis, psychosis, seizure disorder

## Abstract

Autoimmune encephalitis (AE) can masquerade as severe psychosis and is often misdiagnosed as a primary psychiatric disorder, leading to delays in treatment. In cases of acute onset of psychiatric symptoms, it is important to rule out encephalitis, especially when symptoms are accompanied by neurological signs such as seizures.

We report the case of a 22-year-old male who was initially diagnosed with acute psychosis and admitted to an inpatient psychiatry ward. The presence of recurrent seizures raised concern for AE, prompting transfer to the medical intensive care unit. Following a comprehensive workup, he was diagnosed with anti-N-methyl-D-aspartate (NMDA) receptor AE and treated with immunomodulatory therapy, resulting in the resolution of symptoms.

This case highlights the importance of increased clinician awareness for early diagnosis and timely initiation of treatment to improve patient outcomes. We also discuss various diagnostic criteria and scoring systems that may assist clinicians in guiding testing and management of AE.

## Introduction

Autoimmune encephalitis (AE) is an inflammatory condition in which the immune system attacks the brain, leading to symptoms such as confusion, psychiatric changes (e.g., agitation), cognitive decline, and seizures. This condition can be triggered by infections, malignancies, medications, or immunotherapy [1,2].

Diagnosis involves a comprehensive workup, including cerebrospinal fluid (CSF) analysis and the detection of antibodies targeting neuronal receptors such as N-methyl-D-aspartate (NMDA) receptor, contactin-associated protein 2 (CASPR2), leucine-rich glioma-inactivated 1 (LGI1), and gamma-aminobutyric acid (GABA) receptors in both CSF and serum, along with brain imaging [2,3]. The pathophysiology of anti-NMDA receptor encephalitis involves the production of pathogenic antibodies against the NMDA receptor, predominantly affecting the GluN1 subunit. These antibodies disrupt normal brain signaling, leading to brain swelling or encephalitis.

The highest incidence is observed in young adults, with a female predominance. However, the present case involves a 22-year-old male patient who had a rapid onset of behavioral changes, seizures, and multiple emergency department (ED) visits prior to hospitalization, which ultimately led to definitive diagnosis and treatment [3,4]. The criteria proposed by Graus et al. allow for the diagnosis of possible AE prior to antibody confirmation [2].

Early clinical suspicion, use of available diagnostic criteria, a syndrome-based diagnostic approach, and prompt testing and treatment remain key to achieving complete patient recovery. This article highlights the importance of early diagnosis for improved patient outcomes [2-5]. Through this case report, we emphasize that early suspicion should be raised when acute behavioral changes are accompanied by neurological symptoms such as seizures, even in the absence of prodromal features. A rapid syndrome-based diagnostic approach can facilitate the early initiation of immunomodulatory therapy based on clinical suspicion, rather than waiting for antibody confirmation, thereby improving patient outcomes.

## Case presentation

A 22-year-old gentleman was brought to the ED by the police after his parents called for help, as the patient was acting strangely, agitated, running out of the home, and throwing himself violently on the floor, and the family was unable to control his behavior. This was the third ED visit in under two weeks and will be referred to as the index visit in this case presentation.

The patient had no known past medical history until 11 days prior to the index visit, when he was first brought to the ED by his coworkers from a construction site due to a witnessed seizure. CT head was unrevealing, and the patient was discharged home with advice to follow up as an outpatient for a solitary seizure episode.

Three days prior to the index visit, he was again brought to the ED by his coworkers from the construction site due to a witnessed seizure lasting several minutes while he was at work. On presentation to the ED, he reported headache and muscle soreness; he was confused and irritable. No tongue bite or focal weakness was noted. He was admitted overnight; a brain MRI was performed, and showed no acute findings. Electroencephalography (EEG) did not demonstrate epileptiform discharges. He was started on levetiracetam 750 mg twice daily and was discharged.

During the index ED visit, he presented with agitation and an inability to control his behavior. Both neurology and psychiatry were consulted in the ED. Behavioral agitation was suspected to be a potential side effect of levetiracetam; neurology recommended stopping levetiracetam and starting him on lacosamide 100 mg twice daily for seven days, with a plan to increase lacosamide to 150 mg twice daily after seven days. Levetiracetam can cause side effects such as increased irritability, agitation, restlessness, mood swings, aggression, and anxiety. Since the patient developed agitation within three days of starting levetiracetam and there was no other clear etiology for the rapid onset of agitation, it was decided to stop the medication and monitor for improvement in symptoms after discontinuation. Psychiatry recommended inpatient psychiatric admission for acute psychosis, and he was started on divalproex sodium 250 mg twice daily and olanzapine 5 mg twice daily.

While he was admitted to the inpatient psychiatry unit, he missed two doses of all his medications due to his refusal to take them. Later that day, the rapid response team (RRT) was called, as he was found unresponsive for an unknown amount of time. Subsequently, during RRT, he had three more episodes of witnessed seizures with left eye deviation, jerking movements in both upper and lower extremities, and foaming at the mouth. He was administered multiple doses of midazolam. His oxygen saturation dropped from 92% to 54%; he was intubated and admitted to the medical intensive care unit (MICU) due to possible status epilepticus.

On day one in the MICU, he remained on midazolam, fentanyl, propofol, and lacosamide infusion, with severe agitation even on minimal down-titration of medications. Infectious disease was consulted for possible encephalitis/meningitis, and he was empirically started on vancomycin, ceftriaxone, and acyclovir. On day two in the MICU, he underwent a lumbar puncture. CSF study was negative for infection but showed positive oligoclonal bands and elevated protein at 149 mg/dL (Table 1).

**Table 1:** CSF analysis documenting elevated protein and the presence of oligoclonal bands CSF: cerebrospinal fluid; IgG: immunoglobulin G; VDRL: venereal disease research laboratory test; RBC: red blood cell; WBC: white blood cell

CSF findings	Findings	Normal range
Albumin index	2.9	<9
CSF appearance	Clear	Clear
IgG index	0.48	<0.70
IgG synthesis rate	<0.0	0-12 mg/24 hr
IgG CSF	1.8 mg/dL	0.0-8.0 mg/dL
Lactate CSF	1.9 mmol/L	1.1-2.4 mmol/L
Oligoclonal band numbers	7	0-1
Oligoclonal bands CSF	Positive	
Protein CSF	149 mg/dL	15-60 mg/dL
VDRL CSF screen	Non-reactive	Non-reactive
RBC CSF	<2000/µL	<1/µL
WBC CSF	16 cells/dL	0-5 cells/dL
Neutrophils CSF	19%	0-6%
Monocytes CSF	81%	15%-45%

Antibiotics and antivirals were stopped; serum and CSF autoimmune panels were sent. On days three and four in the MICU, EEG was repeated and showed severe diffuse encephalopathy; no epileptiform activity or electrographic seizures were noted.

**Figure 1:**
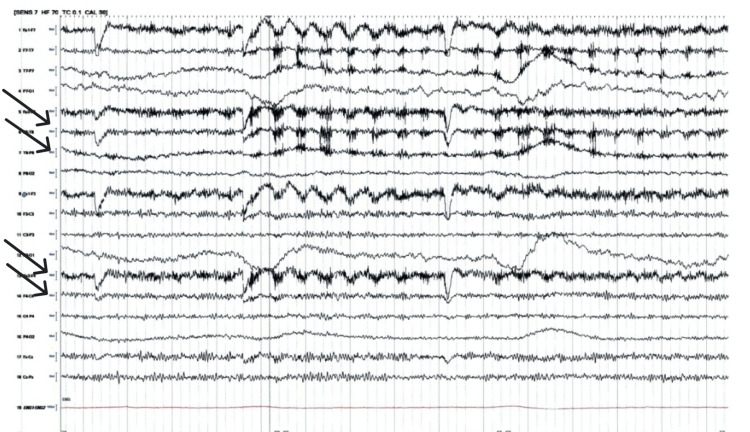
EEG demonstrating an episode of oro-bucco-lingual dyskinesia, consistent with anti-NMDA receptor encephalitis EEG: electroencephalogram; NMDA: N-methyl-D-aspartate

An attempt to minimally wean off sedation led to jerking movements of the lower jaw, suspected to be seizure activity. Continuous EEG was performed; it was indicative of moderate to severe diffuse encephalopathy. Episodes of oro-bucco-lingual dyskinesia were recorded. This pattern is consistent with anti-NMDA receptor encephalitis. No clear epileptiform discharges were noted (Figure 1).

**Figure 2:**
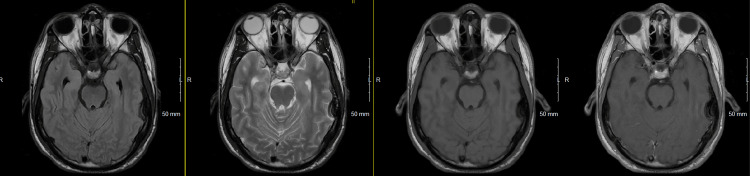
T2 FLAIR, T2 axial, T1, and post-contrast T1 images showing no abnormalities in the bilateral temporal lobes FLAIR: fluid-attenuated inversion recovery

During days five and six in the MICU, pre- and post-contrast MRI of the brain was completed and was unremarkable (Figure 2).

CT imaging of the chest, abdomen, and pelvis, along with a testicular ultrasound to rule out germ cell tumors, was completed and was negative for infectious or malignant processes. Due to high suspicion of AE based on a syndrome-based diagnostic approach and an elevated Antibody Prevalence in Epilepsy and Encephalopathy (APE2) score of eight, our patient was started on high-dose steroids, Solu-Medrol 1 g IV daily for a total of five days.

On MICU day seven, he was continued on high-dose Solu-Medrol. Both serum and CSF AE panel results were positive for NMDA receptor Ab IgG CBA-IFA (Table 2). The patient had an NMDA receptor Ab IgG CBA-IFA titer of 1:160; titers of ≥1:160 are strongly suggestive of autoimmune disease, while lower titers can be non-specific. Serum NMDA receptor Ab IgG CBA-IFA titer was also positive at 1:8.

**Table 2:** CSF result showing positive NMDA receptor Ab IgG NMDA: N-methyl-D-aspartate; Ab: antibody, IgG: immunoglobulin G; CBA: cell-based assay; IFA: indirect fluorescent antibody; CSF: cerebrospinal fluid; PCCS: Purkinje cell cytoplasmic antibodies; ANNA: anti-neuronal nuclear antibodies; AMPA: alpha-amino-3-hydroxy-5-methyl-4-isoxazolepropionic acid; GABA-B: gamma-aminobutyric acid type B; CASPR2: contactin-associated protein-like 2 antibody; LGI1: leucine-rich glioma-inactivated 1; CRMP5: collapsin response mediator protein 5

Test	Result	Reference value
NMDA receptor Ab IgG CBA-IFA, CSF	1:160	<1:1
Paraneoplastic Abs (PCCS/ANNA) IgG, CSF	None detected	None detected
AMPA receptor AB IgG CBA-IFA serum, CSF	<1:1	<1:1
GBBA-BR Ab IgG CBA-IFA serum, CSF	<1:1	<1:1
CASPR2 Ab IgG CBA_IFA serum, CSF	<1:1	<1:1
LGI, Ab IgG CBA-IFA serum, CSF	<1:1	<1:1
CV. Ab IgG CBA-IFA serum, CSF	<1:1	<1:1

**Figure 3:**
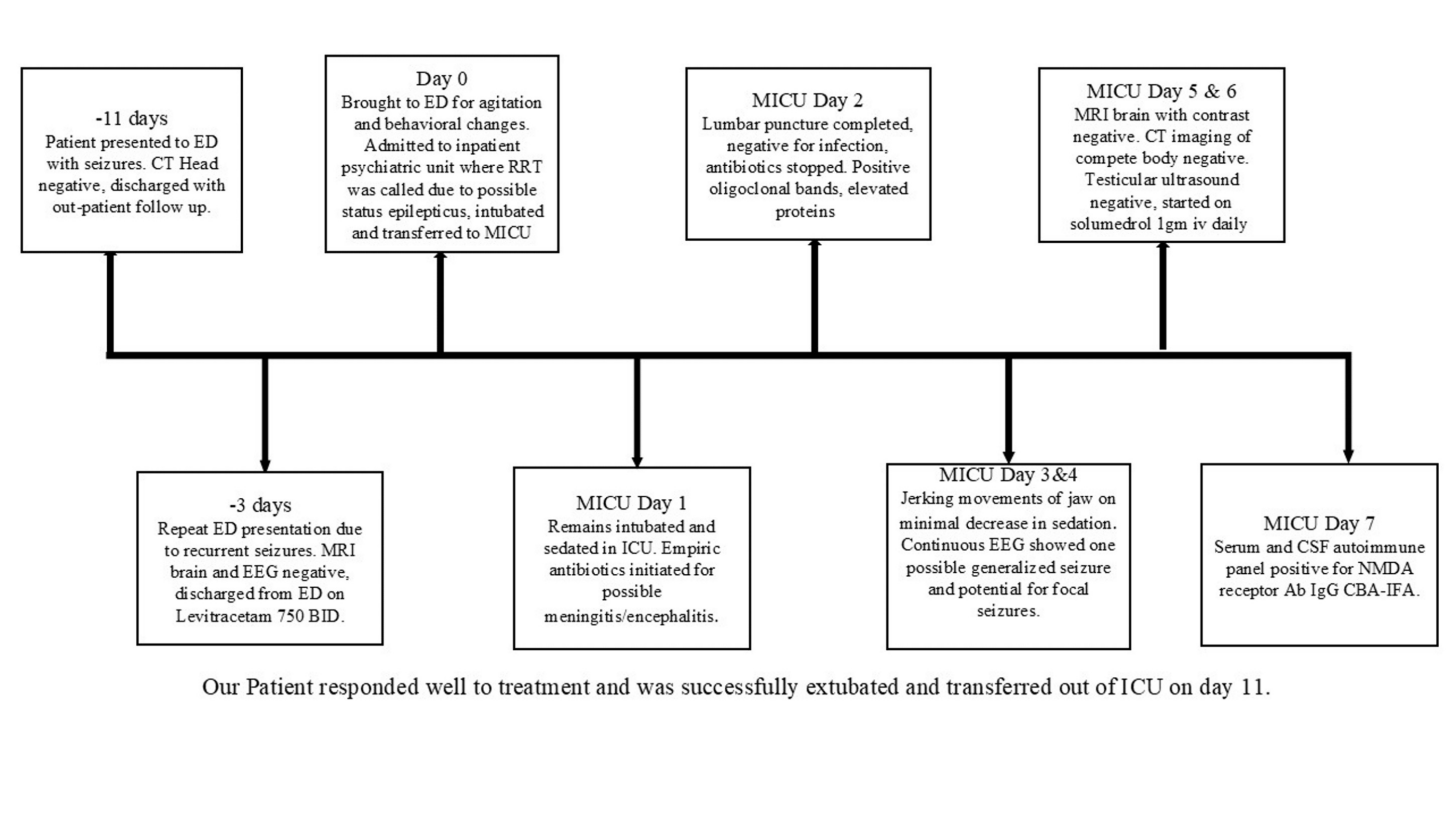
The clinical course of the patient during hospitalization ED: emergency department; CT: computed tomography; RRT: rapid response team; MICU: medical intensive care unit; MRI: magnetic resonance imaging; EEG: electroencephalogram; ICU: intensive care unit; NMDA: N-methyl-D-aspartate; Ab: antibody; IgG: immunoglobulin G; CBA: cell-based assay; IFA: indirect fluorescent antibody; CSF: cerebrospinal fluid

He responded to immunomodulatory therapy, tolerated weaning of sedation with no further seizures, and was successfully extubated and transferred out of the ICU after an 11-day stay. His mental status improved, with no further behavioral symptoms; psychosis and agitation also resolved. He was subsequently discharged to the Acute Rehabilitation Unit (ARU) and then returned home after six weeks in the ARU. We have created a flow diagram representing the patient's hospital course (Figure 3).

At the most recent follow-up, the patient continued to improve, and at three months after ARU discharge, he had resumed construction work with seizure precautions, with no residual neurological or cognitive symptoms reported. Unfortunately, he has not attended subsequent scheduled visits.

## Discussion

Our patient had a delay in the diagnosis of AE, leading to multiple ED visits and admission to the inpatient psychiatry ward instead of a medical ward during the initial course of illness. Use of a syndrome-based approach for diagnosis during the first few ED presentations could have prevented the delay in treatment [2,6].

Syndrome-based diagnosis for anti-NMDA receptor encephalitis means that the initial diagnosis is made based on a specific constellation of clinical symptoms rather than waiting for confirmatory antibody testing results, as prompt treatment is critical for recovery. Using a syndrome-based approach, clinicians can quickly identify key symptoms indicative of AE; this allows differentiation from infections, metabolic disorders, and other neurological conditions that may present similarly. This approach streamlines the diagnostic process, enhances accuracy, and facilitates prompt intervention. The syndrome-based diagnostic approach has also allowed for the development of diagnostic criteria such as the Graus et al. criteria, which are beneficial tools for early diagnosis and treatment, ultimately leading to improved patient outcomes.

As emphasized by Graus et al., it remains imperative to use a comprehensive, syndrome-based clinical approach for diagnosing AE. Following this approach led to the initiation of treatment in our patient prior to obtaining antibody test results [2]. The Graus et al. criteria, published in 2016, are standardized, evidence-based guidelines for diagnosing AE, including anti-NMDAR and antibody-negative cases. They allow clinicians to initiate immunotherapy early based on rapid symptom onset (less than three months), specific neuropsychiatric features, and, if available, MRI/EEG findings, without waiting for antibody results.

Turnaround time for serum and CSF antibody testing can also cause delays in treatment, which was not the case in our patient [7]. Immunomodulatory treatment was started prior to receiving antibody test results due to high clinical suspicion and the use of a comprehensive, syndrome-based approach, leading to complete recovery in our patient [2-8].

Anti-NMDA receptor encephalitis is a disorder that usually affects young adults and children, with a female predominance in young adults [3,4]. The disorder is associated with a predictable set of symptoms that combine to form a characteristic syndrome [7]. Many patients present with prodromal symptoms such as headache, fever, or viral illness, none of which were reported in our patient, potentially contributing to the initial delay in diagnosis. Yadav et al. reported a case that was initially treated as tubercular meningitis due to the presence of fever, headache, and the high prevalence of tuberculosis in the patient’s geographical location [7].

Prodromal symptoms are followed by psychiatric manifestations such as anxiety, agitation, bizarre behavior, hallucinations, delusions, disorganized thinking, and psychosis [4]. Additionally, sleep disturbances, memory deficits, seizures, and decreased level of consciousness may occur. Our patient presented with agitation, memory deficits, and new-onset seizures [8].

The most important diagnostic tests include CSF studies, EEG, and MRI of the brain. The confirmatory test is the presence of antibodies related to AE in CSF, serum, or both. CSF analysis may show elevated protein and oligoclonal bands, both of which were noted in our patient. EEG typically shows infrequent epileptic activity but often demonstrates slow and disorganized activity that does not correlate with abnormal movements, as seen in our patient. In less than 30% of cases, EEG demonstrates a pattern called extreme delta brush, which can assist in diagnosis but is associated with more prolonged illness and poorer prognosis [9].

Brain magnetic resonance imaging (MRI) is often normal but may show high-intensity signals in the medial temporal lobes or cerebral atrophy [10]. Positron emission tomography (PET) reportedly shows a characteristic increase in the frontal-occipital gradient of cerebral glucose metabolism, which correlates with disease severity; however, this test may not be routinely available. The diagnosis of anti-NMDA receptor encephalitis is confirmed by the detection of immunoglobulin G (IgG) antibodies against the GluN1 (also known as NR1) subunit of the NMDA receptor [2-11]. During the initial illness, CSF testing is more sensitive than serum testing.

Differential diagnoses that were considered included primary psychotic disorder, which was ruled out due to associated neurological symptoms and rapid onset, and substance-induced psychosis, which was ruled out with a negative urine drug screen. Herpes simplex virus (HSV) encephalitis was ruled out with a negative HSV polymerase chain reaction (PCR) in CSF.

Initiation of treatment based on a syndrome-based diagnostic approach is crucial, given antibody testing results may not be immediately available and turnaround time can vary significantly, typically taking anywhere from a few days (4-6 days) to up to three weeks [4-12]. It is recommended that all patients be screened at least once for cancer, as certain cancers, including germ cell tumors, are known to trigger the immune system to attack the brain. Our patient had negative imaging studies, and malignancy was ruled out prior to initiating treatment [6]. It is important to look for red flags early in disease onset; we have included Table 3 to show red flag symptoms for AE in acute psychiatric presentations.

**Table 3:** Red flags of possible AE in patients with neuropsychiatric disorders AE: autoimmune encephalitis

Red flags of possible AE in patients with neuropsychiatric disorders
New onset of acute psychiatric episode (acute mania, catatonia, psychosis)
Rapidly progressive short-term memory and cognitive decline
Unexplained new-onset intractable epilepsy or status epilepticus
New-onset movement disorders (e.g., dyskinesias and dystonia)
Deterioration, relapses, or emergence of new neurological and/or neuropsychiatric symptoms following confirmed or presumed viral illness
Infectious-like prodromal illness
Strong personal or family history of autoimmune disorders
Recent diagnosis of a neoplasm

Various scores can be used by clinicians to guide testing and treatment in AE. The APE2 score is designed to predict the likelihood of neural-specific antibodies in patients with new-onset seizures or rapidly progressive cognitive changes (Table 4). Key features considered in the score include: new-onset, rapidly progressive mental status changes or new-onset seizures, neuropsychiatric changes, faciobrachial dystonic seizures, seizures refractory to at least two anti-seizure medications, brain MRI findings of encephalitis, and a systemic cancer diagnosis within the past five years of neurological symptom onset. A score of ≥4 indicates a high likelihood of neural-specific antibodies associated with autoimmune encephalopathy or epilepsy. This score helps guide the decision to order autoimmune evaluations in serum and CSF for antibody testing. Our patient had an elevated APE2 score of 10, which helped direct CSF and serum antibody testing.

**Table 4:** APE2 score APE2: antibody prevalence in epilepsy and encephalopathy; CSF: cerebrospinal fluid; MRI: magnetic resonance imaging; FLAIR: fluid-attenuated inversion recovery

APE2 score	
New-onset, rapidly progressive mental status changes that developed over 1-6 weeks or new-onset seizure activity(within 1 year of evaluation)	+1
Neuropsychiatric changes: agitation, aggressiveness, and emotional liability	+1
Autonomic dysfunction (sustained atrial tachycardia or bradycardia, orthostatic hypotension, hyperhidrosis, persistently labile blood pressure, ventricular tachycardia, cardiac asystole, or gastrointestinal dysmotility)	+1
Viral prodrome (rhinorrhea, sore throat, and low-grade fever) to be scored in the absence of underlying systemic malignancy within 5 years of neurological symptom onset	+2
Faciobrachial dystonic seizures	+3
Facial dyskinesias, to be scored in the absence of faciobrachial dystonic seizures	+2
Seizure refractory to at least two anti-seizure medications	+2
CSF findings consistent with inflammation (elevated CSF protein >50 mg/dL and/or lymphocytic pleocytosis >5 cells/mcl	+2
Brain MRI suggesting encephalitis (T2/FLAIR hypersensitivity restricted to one or both medial temporal lobes, or multifocal in grey matter, white matter, or both, compatible with demyelination or inflammation)	+2
Systemic cancer diagnosed within 5 years of neurological symptom onset(excluding cutaneous squamous cell carcinoma, basal cell carcinoma, brain tumor, or cancer with brain metastasis	+2

The Clinical Assessment Scale in Autoimmune Encephalitis (CASE) score is a clinical tool specifically developed to monitor the severity of AE. This score evaluates nine motor and non-motor symptoms, including psychiatric manifestations, seizures, and language deficits. It ranges from 0 to 27, with higher scores indicating more severe disease. This score is used for monitoring disease severity and treatment response. The APE2 and CASE scores complement each other and are valuable in guiding early immunotherapy, improving diagnostic accuracy, and optimizing patient management in AE.

Treatment of anti-NMDA receptor encephalitis consists of tumor removal, if associated, and immunomodulatory therapy [8]. The initially recommended treatments include glucocorticoids, intravenous immunoglobulin, or therapeutic plasma exchange. For patients who do not respond adequately to the above treatments, second-line therapy with rituximab (preferred second-line treatment), ofatumumab, other B-cell-depleting therapies, as well as tocilizumab, is reasonable to consider [4].

## Conclusions

The case supports the importance of considering neurological causes in young patients with new-onset psychiatric symptoms. Rapid-onset neurological symptoms such as seizures, involuntary movements, cognitive decline, and altered mental status should raise concern for AE, especially in younger patients. Use of the APE2 scoring system helps predict the likelihood of antibody positivity in cases of encephalitis and encephalopathy and helps guide initiation of treatment without waiting for antibody testing results. Our patient had a high APE2 score that prompted further evaluation for AE. The Graus et al. criteria are a practical, syndrome-based diagnostic approach to AE, which helped us initiate prompt immunotherapy prior to receiving antibody test results.

The recommendation for early treatment based on clinical suspicion is consistent with current guidelines. The improvement after immunomodulatory therapy aligns with the reported clinical course. Early treatment has been shown to result in significant improvement and even full recovery in such cases. It is highly encouraged to initiate treatment based on clinical symptoms before definitive results are available to improve patient outcomes.
